# A Technology Selection Support Model in Industry 4.0 for Quality Assurance and Traceability in the Production of Waste-Derived Functional Materials

**DOI:** 10.3390/ma19163360

**Published:** 2026-08-07

**Authors:** Andrzej Pacana, Miłosz Pilch, Małgorzata Ulewicz

**Affiliations:** 1 Faculty of Mechanical Engineering and Aeronautics, Rzeszow University of Technology, Al. Powstancow Warszawy 12, 35-029 Rzeszow, Poland; 2 Safran Aircraft Engines Poland Sp. z o.o., Południowa 23, 39-120 Sędziszów Małopolski, Poland; 8pilchu@gmail.com; 3 Faculty of Civil Engineering, Czestochowa University of Technology, ul. J.H. Dabrowskiego 69, 42-201 Czestochowa, Poland; malgorzata.ulewicz@pcz.pl

**Keywords:** functional materials, circular economy, waste valorization, recycling, quality assurance, traceability, Industry 4.0, decision model, production engineering

## Abstract

The circular economy increases the importance of quality and traceability in functional materials produced from industrial, municipal, and agricultural waste. However, the selection of Industry 4.0 (I4.0) technologies in waste valorization remains unsystematic and weakly linked to material and process requirements. This study develops a decision-support model for selecting I4.0 solutions for quality assurance and traceability in the production of waste-derived functional materials. The model was grounded in a two-track scoping review reported according to PRISMA-ScR. Track A mapped the functions of I4.0 technologies in quality control and manufacturing traceability, while Track B identified challenges related to waste-derived biopolymers, composites, sorbents, catalysts, and nanomaterials. Among 322 primary material studies, microstructure, morphology, and porosity dominated (69.9%), whereas traceability was underrepresented and requires further validation. In Track A, 629 studies were unambiguously mapped, with inspection and monitoring as the dominant functions. Material-specific challenges were then linked to required functions and candidate technologies, which were ranked by their fit to the decision situation and literature support. The resulting model covered 48 material situations and 95 function–technology pairs. It supports technology selection based on material category, waste type, process stage, and quality or traceability requirements rather than technology availability alone. The proposed approach may be extended to waste-derived materials for other application sectors, including construction and transport, subject to sector-specific, material-specific, and industrial validation.

## 1. Introduction

As the circular economy continues to develop, increasing importance is placed on converting industrial, municipal, and agricultural waste streams into functional materials with reproducible properties and documented provenance [1,2]. Waste-derived functional materials, including biopolymers, composites, sorbents, catalysts, and nanomaterials, are increasingly regarded as value-added products rather than merely as by-products of waste management processes [3,4]. Their production, however, involves challenges related to ensuring adequate quality, because the properties of the final material may depend strongly on feedstock variability. This variability may concern, among other factors, feedstock origin, composition, contamination level, moisture content, and prior processing history [5,6]. Consequently, maintaining consistent quality may be more difficult than for materials produced from feedstocks of standardized origin. Depending on the material category, quality criteria may include chemical composition and purity, morphology, porosity, specific surface area, mechanical properties, thermal stability, catalytic activity, sorption capacity, phase dispersion, and batch-to-batch reproducibility [3,5,6].

In parallel, the importance of traceability of both the material and the data associated with its production is increasing, particularly in the context of the circular economy, material reuse, and digital product passports [7,8]. In this article, traceability is operationally understood as the ability to link the final material and its properties to the origin of the waste stream, process conditions, laboratory test results, batch history, and quality documentation. For materials obtained from different waste streams, the ability to reconstruct these relationships is particularly important for assessing the causes of variability and documenting material conformity with established requirements.

I4.0 technologies can support these tasks at different stages of the process [9,10]. The use of IoT (Internet of Things)-based monitoring and traceability also requires reliable device identification and data security, for which hardware-level solutions such as physically unclonable functions may provide additional support [11]. SCADA (Supervisory Control and Data Acquisition) systems enable the monitoring of process parameters and external conditions [10,12], whereas artificial intelligence and machine learning can support predictive quality analysis [13]. Machine vision systems are used for automated inspection [14], while digital twins support simulation, process-state representation, and optimization [15]. In turn, LIMS (Laboratory Information Management System), MES (Manufacturing Execution System), and ERP (Enterprise Resource Planning) systems can support the integration of laboratory, process, and administrative data, documentation management, and the recording of operational histories [16]. RPA (Robotic Process Automation) can complement this layer by automating repetitive, rule-based data and document flows between existing systems [17].

Previous studies have addressed both waste-derived functional materials and digital transformation in quality, traceability, and the application of individual I4.0 technologies. However, these research streams focus on different levels of the problem and only partially address the need to select technologies for a specific material situation. Table 1 summarizes representative research directions directly related to the scope of the present study.

The comparison indicates that the literature provides a basis for the individual components of the problem under investigation; however, it does not offer an integrated approach linking the category of waste-derived material, feedstock source, process stage, and quality or traceability requirement with an I4.0 function and a structured technology selection process. The present study seeks to address this gap.

The literature on I4.0 technologies in quality control and quality assurance is extensive, as is the body of research on the production of waste-derived functional materials. However, these two research streams have largely developed separately. An approach is lacking that would link material-specific quality and traceability challenges with I4.0 technology functions and subsequently use this relationship to support the selection of technological solutions. As a result, technology selection may be based on the general capabilities of a given solution without sufficient reference to the material type, feedstock origin, process stage, and specific quality-related problem. The aim of this article is to develop a model supporting the selection of I4.0 technologies for quality assurance and traceability in the production of waste-derived functional materials. The proposed model is recommendation-oriented rather than purely descriptive. It integrates material category, waste type, process stage, quality and traceability requirements, and I4.0 functions, and subsequently ranks candidate technologies by taking into account their fit to the analyzed situation, the literature support status, and the degree of grounding of the function–technology relationship. The scope of the study covers five categories of waste-derived functional materials: biopolymers, composites, sorbents, catalysts, and nanomaterials, as well as I4.0 technology functions related to monitoring, prediction, inspection, simulation and optimization, data integration, documentation and reporting, and maintenance of an audit trail.

The scope of the study was defined by four research questions:

RQ1: What quality and traceability challenges occur across the individual categories of waste-derived functional materials, and how are they distributed in the analyzed literature?

RQ2: What functions do I4.0 technologies perform in ensuring quality and traceability in manufacturing processes, and which technology–function relationships are represented in the literature?

RQ3: Which I4.0 technologies can be recommended for the individual categories of functional materials and the identified quality or traceability challenges?

RQ4: To what extent do the model recommendations have direct counterparts in the literature, and what is the nature of their literature support?

To identify and organize the input data for the model, a two-track scoping review of the literature was conducted and reported in accordance with the PRISMA-ScR guidelines. Track A covers I4.0 technologies applied to quality control, quality assurance, and traceability in manufacturing processes. Track B focuses on quality and traceability challenges concerning five categories of waste-derived functional materials: biopolymers, composites, sorbents, catalysts, and nanomaterials. The two tracks are analyzed independently, and their results are integrated only at the synthesis stage. The identified material challenges are then assigned the required I4.0 functions, which are subsequently linked to technologies and used to construct the decision–support matrix. The scoping review is therefore not an end in itself, but provides the basis for developing and applying the model.

The contribution of the article comprises three principal elements. The first is the identification and structuring of quality and traceability challenges specific to waste-derived functional materials. The second is the classification of I4.0 technologies according to the functions they perform in quality assurance and traceability, rather than solely according to technology names. The third is a decision–support matrix that links material requirements with I4.0 functions and potential technological solutions, ranks candidates according to their fit to the situation, and distinguishes between direct, functional, and exploratory forms of literature support.

## 2. Materials and Methods

### 2.1. Study Design and Scoping Review Strategy

The study was designed as an article focused on the development of a technology selection support model, with a two-track scoping review of the literature providing its evidence base. The review was conducted to map two distinct areas of knowledge required for the development of the model: the functions of Industry 4.0 technologies and the quality and traceability challenges associated with waste-derived functional materials. The review procedure was reported in accordance with the PRISMA-ScR guidelines [21,22]. The review is not an end in itself, but serves to identify and organize the data required to construct the model. The research procedure comprises five principal stages: (i) identification of quality and traceability challenges specific to the materials under analysis, (ii) determination of I4.0 functions relevant to quality assurance and traceability, (iii) coding and classification of the collected evidence, (iv) development of the decision–support matrix, and (v) evaluation of the proposed model in terms of logical consistency and agreement with the available literature evidence.

Track A covers studies on the application of I4.0 technologies to quality control, quality assurance, and traceability in manufacturing processes. Its primary purpose is to determine the functions performed by individual technologies and to assess the degree to which these functions are established in the literature. Track B has a different scope. It focuses on quality and traceability problems occurring in five categories of waste-derived functional materials: biopolymers, composites, sorbents, catalysts, and nanomaterials. The results of the two tracks are integrated only at the synthesis stage, during which the identified material challenges are assigned corresponding I4.0 functions.

The differences between the tracks arise from their distinct objectives. In Track A, both primary studies and review articles play an important role because existing classifications and syntheses of research on I4.0 in the quality domain provide a basis for developing a taxonomy of technological functions. In Track B, by contrast, the main corpus was restricted to primary studies so that the identified material challenges would arise directly from research concerning the production, characterization, or application of specific materials. Review articles in Track A were subjected to the same functional coding scheme as primary studies because the purpose of this track was to map technology–function relationships rather than to assess implementation effectiveness. Since the unit of analysis was the document, the resulting frequency distributions describe the structure of the analyzed publication corpus rather than the number of independent technology implementations.

The differing nature of the two research fields also influenced the design of the search strategies. The literature on I4.0 applications in manufacturing quality is extensive; therefore, Track A adopted a high-precision strategy with strong reliance on title-field searching. In Track B, a broader search across titles, abstracts, and keywords was necessary because information on feedstock variability, material properties, or reproducibility problems often does not appear in the publication title alone.

After completion of the two main tracks, an auxiliary AB verification set was additionally used, targeting publications that directly linked the analyzed waste-derived functional materials with I4.0 technologies in the context of quality or traceability. The AB set did not constitute a separate review track and was not involved in the identification of material challenges or in the construction of the technology–function map. It was used at the recommendation evaluation stage to determine whether directly corresponding applications reported in the literature existed for selected relationships.

The search strategy was based on three conceptual blocks. In Track A, these comprised I4.0 technologies, objectives related to quality and traceability, and the manufacturing context. The first block included, among others, IoT, SCADA, digital twins, machine learning and deep learning, machine vision systems, RPA, and blockchain. The second block contained terms referring, among others, to quality control, quality assurance, defect detection, fault detection, process monitoring, predictive quality, traceability, and audit trail. The third block included terms indicating the manufacturing environment, such as manufacturing, production process, industrial process, and fabrication.

For Track B, an analogous three-block combination logic was applied; however, the blocks corresponded to material category, waste origin, and quality or traceability challenges. Synonyms and related terms within each block were combined using the OR operator, whereas the AND operator was used between blocks. The material block was constructed to cover the five categories analyzed in the article: biopolymers, composites, sorbents, catalysts, and nanomaterials. The waste-origin block included, among others, the terms waste-derived, waste-based, residue-derived, by-product-derived, agricultural residues, agro-waste, food waste, agro-industrial waste, by-products, and valorisation/valorization. The quality-and-traceability block, in turn, included terms related, among others, to quality control, quality assurance, feedstock variability, reproducibility, consistency, batch-to-batch variation, standardisation/standardization, heterogeneity, traceability, chain of custody, provenance, batch tracking, and material passports.

The searches were conducted in the Scopus and Web of Science databases. Only English-language publications were eligible for analysis. Articles and conference papers were included in the main corpus, with review articles additionally included in Track A. The results from both databases were subsequently merged and deduplicated. In Web of Science, fields corresponding to the query scope adopted in Scopus were used, in particular the TS field as the equivalent of topic searching covering titles, abstracts, and keywords. In Track B, the full TS query was treated as the reference corpus, whereas the subset restricted to Web of Science Categories = Materials Science, Multidisciplinary was used as the working corpus. Functionally, this approach corresponded to the use of the materials-oriented MATE subset in Scopus. It should be emphasized that the two subsets are not direct equivalents of one another. Scopus and Web of Science use different subject-classification systems, namely ASJC and Web of Science Categories, respectively. The results obtained from the two databases were therefore treated as two independent sources of records and were combined only during the merging and deduplication stage.

### 2.2. Eligibility Criteria, Study Selection, and Data Coding

The eligibility criteria for Track B were developed based on the PCC framework (Population, Concept, Context). The Population comprised waste-derived functional materials belonging to one of five categories: biopolymers, composites, sorbents, catalysts, and nanomaterials. The Concept component covered quality and traceability challenges, in particular feedstock variability, composition, purity, morphology, specific surface area, porosity, mechanical properties, thermal stability, activity, selectivity, batch-to-batch reproducibility, documentation completeness, and traceability. The Context encompassed the production, synthesis, processing, and valorization of waste-derived materials. A study was included in the analysis if it met all three of the following conditions: (i) it concerned at least one of the five material categories covered by the study, (ii) the analyzed material was produced from waste, residue, or a by-product, and (iii) the publication addressed at least one quality or traceability challenge.

Given the size of the corpus, the primary data extraction in Track B and the coding of I4.0 functions in Track A were conducted on the basis of titles, abstracts, and keywords. Full texts were analyzed in cases that could not be classified unambiguously based on the available metadata and abstract, as well as during the in-depth extraction of model situations and the development of the model application example. This extraction scope enabled the analysis of a large corpus, but it may have resulted in the omission of information reported exclusively in full texts; this limitation is addressed in Section 4.3 (Limitations and Further Validation).

The challenges identified in Track B were coded according to seven operational categories: C1, feedstock variability and heterogeneity; C2, composition, purity, and contamination; C3, microstructure, morphology, and porosity; C4, control and prediction of properties; C5, reproducibility, standardization, and scale-up; C6, stability, durability, and degradation; and C7, traceability, provenance, and authenticity. Each study was assigned one primary challenge used in the core RQ1 map, whereas secondary challenges were recorded separately. Category C4 was applied only when control, modeling, optimization, or prediction of properties constituted the core focus of the study; material characterization alone did not provide a basis for assignment to this category.

For I4.0 technologies, coding was not limited to the name of the solution. The key task was to determine the function performed by a given technology in the analyzed application. The following functions were distinguished: monitoring, prediction, inspection, simulation and optimization, data integration, documentation and reporting, and maintenance of an audit trail. This approach was necessary because the same technology may perform different tasks depending on the material, process stage, and type of quality requirement. For example, AI can support predictive quality analysis, machine vision systems for automated inspection, SCADA process monitoring, and digital twin simulation and optimization. In turn, RPA-based workflows and LIMS and MES systems can support documentation, data integration, and traceability. The technology function thus provides a common level of description that enables subsequent integration of the results from the two tracks.

The same set of functions was used to code records in Track A. Each record could be assigned to one or more functions: monitoring, prediction, inspection, simulation and optimization, data integration, documentation and reporting, and maintenance of an audit trail. This made it possible to determine which functions are most frequently performed by individual technologies and the extent to which they are represented in the analyzed literature. The coding of I4.0 functions was carried out by one researcher using the adopted classification scheme. In cases that could not be classified unambiguously based on the title, abstract, and keywords, the full text of the publication was examined. Only records for which the function assignment was unambiguous were included in the technology–function map. The functions constitute a common level of description for both review tracks. In Track B, challenges arising from material properties, waste type, and process conditions are identified, whereas Track A provides information on technologies capable of performing specific quality- and traceability-related functions. The integration therefore proceeds in two stages: first, the required function is assigned to the material challenge, and then the function is linked to technologies for which such an application is supported by evidence identified in Track A. In formal terms, this corresponds to a transition from the material problem to the function, and subsequently from the function to the technology.

A literature support status was additionally assigned to each recommendation. Status A was assigned when a publication directly corresponding to the analyzed situation and applied technology was identified in the auxiliary AB set. Status B indicated the absence of such a direct counterpart while both links in the synthesis remained supported: Track B documented the material challenge, and Track A documented the relationship between the required function and the technology. Status C was applied in cases where at least one mapping link required analogy or extrapolation, or had limited support. This status describes the nature of the literature support for the recommendation and does not constitute an assessment of the methodological quality of publications or a validation of effectiveness. Table 2 presents the functional layer of the proposed model.

The six layers encompass seven functions because the final layer combines documentation and reporting with maintenance of an audit trail. The table does not yet constitute the complete decision matrix because it does not include all model input variables, such as material category, waste type, process stage, specific quality or traceability challenge, the literature support status, or the final recommendation. It does, however, define the mechanism for transitioning from a material problem to the required I4.0 function and subsequently to technologies that may serve as the primary or supporting solution. In the operational version of the model, the set Ψ(f) was determined based on the two technologies most frequently co-occurring with function f in Track A records. The list presented in the table therefore defines the space of possible solutions but does not yet determine the final recommendation for a specific material case.

The response to RQ1 was based on mapping Track B studies according to material category and the primary quality or traceability challenge. RQ2 was analyzed on the basis of the technology–function relationships identified in Track A. RQ3 concerned the stage at which the two tracks were integrated within the recommendation model. RQ4 was analyzed based on the literature support status of the recommendations and the presence of directly corresponding cases in the auxiliary AB set.

To organize the data used to develop the model, Table 3 presents the structure of both scoping review tracks. The table shows the successive stages of data processing, from records retrieved from bibliographic databases, through the datasets subjected to coding, to the material situations and function–technology pairs used in the model.

This structure distinguishes three types of input data used in the model. Track B describes the material situation and provides the basis for assigning the required function; Track A identifies the technologies capable of performing that function. The integration of these levels results in a set of function–technology candidates, whose evaluation and ranking procedure are presented in the following subsection.

### 2.3. Model Construction, Recommendation Support Status, and Evaluation Procedure

The construction of the model was based on three assumptions. First, technology selection should result from the nature of the material problem and the required function rather than from the mere availability of a solution. Second, the fit of a technology to a decision situation is assessed independently of the directness of its literature support. Third, the model output is intended to provide decision support and does not replace an assessment of technological feasibility or validation under the conditions of a specific industrial plant.

The model integrates four descriptors of the decision situation: material category, waste type, process stage, and quality or traceability requirement. The output is a technology recommendation comprising a primary solution and, where justified, supporting technologies. The I4.0 function constitutes an intermediate level between the identified challenge and a specific technology. This means that a material challenge is first mapped to the function required in a given situation and only then to technologies capable of performing that function. Such an approach makes it possible to separate the need arising from material and process characteristics from the selection of a specific technological solution. In the operational analysis of 48 situations, each situation was assigned one primary function that best corresponded to the dominant challenge. This provided a consistent basis for comparing situations and constructing the recommendation matrix. The formal structure of the model also allows supporting functions; an extended variant of such mapping is presented in the example in Section 4.

**Figure 1:**
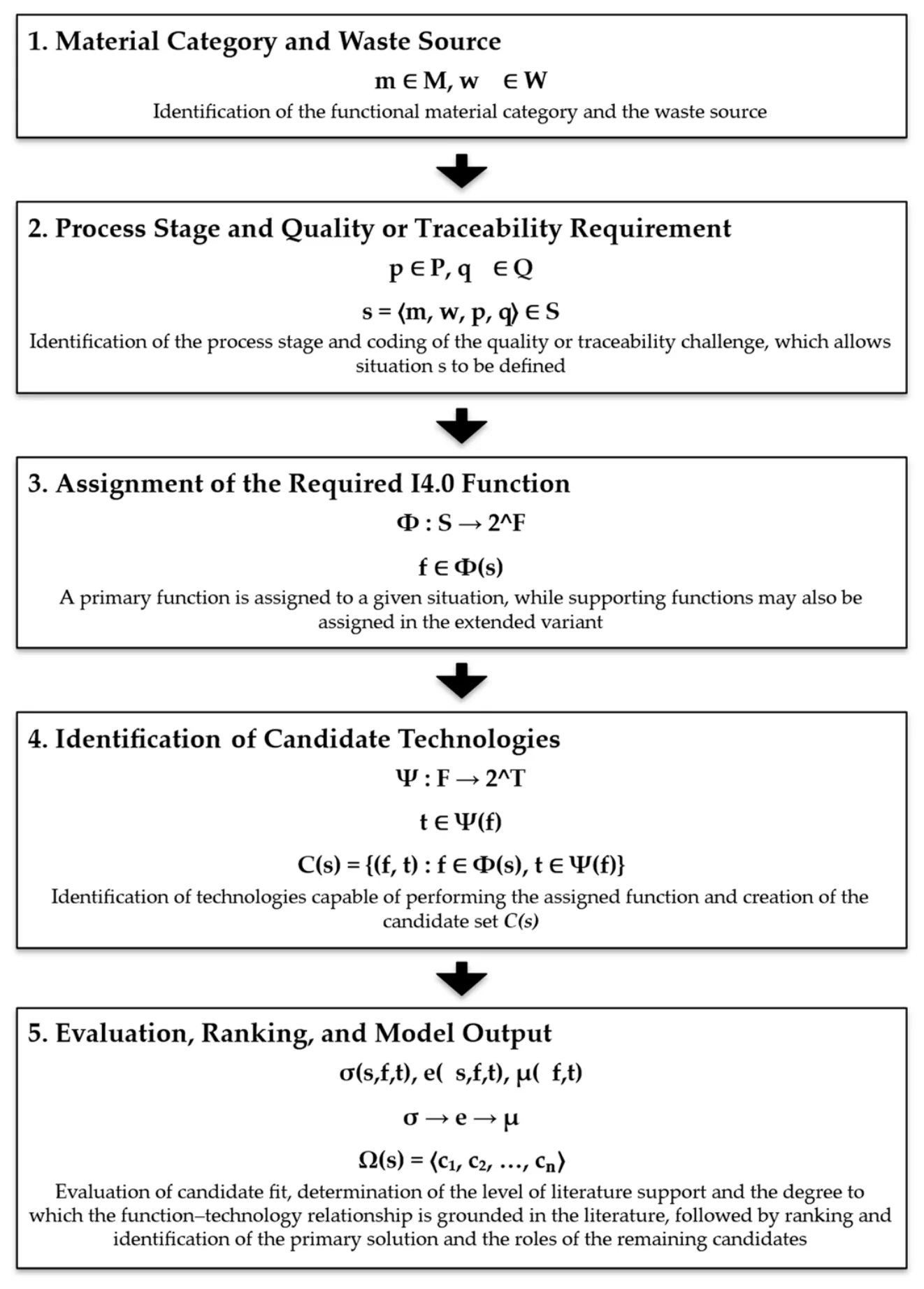
Reasoning chain of the Ω decision–support model.

Figure 1 presents the successive stages of the Ω model. The starting point is a description of the decision situation, including the material category, waste source, process stage, and the quality or traceability requirement. On this basis, the required Industry 4.0 function is identified, followed by the technologies capable of performing it.

The procedure follows the five stages shown in Figure 1, while its detailed mathematical formalization is provided in Appendix A.

The decision situation is described as:

(1) s= <m,w,p,q>

where m denotes the material category, w the waste type or source, p the process stage, and q the quality or traceability requirement, or a combination thereof. For a situation described in this manner, the model first identifies the required function f and then assigns a technology t to that function. The output is an ordered set of recommendations comprising a primary technology and supporting solutions, together with information on literature support status, degree of fit, and the grounding of the function–technology relationship in the literature.

The fit of a recommendation to the situation is described by the score σ. Four components are assessed: compatibility with material properties, process stage, quality requirement, and traceability requirement. A value of 0 denotes no fit, 1 partial fit, and 2 full fit of technology t, performing function f, to the given dimension of situation s. The maximum score is 8 when both quality and traceability requirements are active. When only one of these dimensions is active, the other component is assigned a value of 0, and the maximum score is 6. To enable comparisons between situations, a normalized score was additionally calculated:

(2) $$\sigma_{N}\left(s,f,t\right)=\frac{\sigma (s,f,t)}{\sigma_{max}(s)}$$

The normalized score ranges from 0 to 1. It does not alter the ranking within a decision situation because the same value of $\sigma_{max}$ applies to all candidates considered for that situation. Raw σ values are used to rank candidates within the same decision situation, whereas $\sigma_{N}$ allows the level of fit to be compared across situations. Detailed scoring criteria are provided in Appendix A.

The formalization of the model serves primarily to provide an unambiguous representation of the recommendation process: from the problem situation, through the required function, to the technology and the basis for its selection. It is not treated as a separate optimization algorithm or as a substitute for multi-criteria decision–support methods. Its purpose is to make it possible to trace the basis on which a given technology was assigned to a specific problem. An example of the model operation is presented in Section 4. The fit-component scores were assigned based on the material, process, and functional compatibility of the solution with the analyzed situation. The fit score was determined independently of the literature support status e; status A, B, or C was not used as a criterion for assigning fit points.

Each recommendation is described by three distinct characteristics. The score σ specifies the fit of the function–technology pair to a particular situation. Status e describes the nature of the literature support for the recommendation, whereas indicator μ refers to the degree of grounding of the function–technology relationship in the Track A literature. These characteristics serve different purposes and are not treated interchangeably. Recommendations are ranked first according to their fit to the analyzed situation. Where the σ score is the same, a candidate with a more direct literature correspondence is ranked higher, following the order A, B, C. This secondary rule reflects the inferential distance between the recommendation and the available literature, rather than the methodological quality, number of supporting studies, or technology effectiveness. Indicator μ serves as the next auxiliary criterion. This ranking procedure prevents a technology that is widely represented in the literature from achieving a high position despite a poor fit to the specific material problem. In cases where candidates obtained identical values of σ, e, and μ, they were treated as equivalent; no additional arbitrary tie-breaking criterion was applied. The principles for interpreting literature support statuses are summarized in Table 4.

The literature support status of a recommendation, e, does not constitute an assessment of the methodological quality of individual publications or of the overall strength of evidence in the sense used in research quality-assessment systems. It indicates only the degree of directness of the literature support for a given recommendation. It should therefore be distinguished from both the fit score σ and the indicator μ: e describes the directness of the literature basis, σ the fit to the decision situation, and μ the degree of grounding of the function in the Track A literature. Status A/B/C does not represent a hierarchy of study quality or a measure of technology effectiveness. It serves only to distinguish recommendations with a direct counterpart in the literature from those based on functional synthesis or extrapolation.

The evaluation of the model included verification of its internal consistency and assessment of the agreement between the recommendations and cases documented in the literature. In the first stage, it was verified whether each recommendation could be reproduced on the basis of the defined input situation, the assigned function, the technology-selection rule, and the adopted literature support status of the recommendation. The purpose of this stage was to identify recommendations that did not follow from the explicit rules of the model or were inconsistent with the coded challenge. A complementary assessment involved verifying the literature consistency of selected recommendations against cases collected in the auxiliary AB set. Its purpose was to determine whether recommendations generated from the synthesis of Tracks A and B had directly corresponding application examples in the literature. Such agreement was not interpreted as validation of the effectiveness of the model or the technologies, because the presence of a single literature case does not establish the validity of a recommendation in a new situation. The AB set was not used to answer RQ1 or to construct the technology–function map. Its role was limited to determining the direct-support status of selected recommendations.

## 3. Mapping and Model Development Results

The results are presented according to the successive stages of model development. First, the map of Industry 4.0 technology functions developed in Track A is presented, followed by the map of challenges associated with waste-derived functional materials obtained in Track B. The final part of the section presents the integration of both layers and the resulting model recommendations. The auxiliary AB set was used solely to determine the type of literature support for selected recommendations.

### 3.1. Results of Track A: Functions of Industry 4.0 Technologies

In Track A, the searches conducted in the Scopus and Web of Science databases yielded a total of 1220 records. After merging the results and removing duplicates, 752 studies remained. Four records were excluded at the title and abstract screening stage, one full text could not be retrieved, and 29 publications were excluded after further assessment for documented reasons. Ultimately, 718 studies were included in the Track A synthesis. For 629 of these, it was possible to unambiguously determine the relationship between a technology and the function it performed. Within this subset, inspection was identified most frequently, accounting for 264 studies (42.0%), followed by process monitoring, represented by 198 studies (31.5%). Prediction accounted for 80 studies (12.7%), while simulation and optimization were identified in 41 studies (6.5%). Audit trail functions were much less frequently represented (38; 6.0%), as were data integration (6; 1.0%) and documentation and reporting (2; 0.3%). This tendency is further confirmed by classification according to the primary application dimension: approximately 93% of the mappable studies concerned quality, whereas traceability and the combined treatment of quality and traceability each accounted for approximately 3.5% of the corpus.

The degree of grounding of individual functions in the literature, denoted by the indicator μ, was also assessed. Among the 629 mappable studies, 554 were assigned to the emerging level (μ=2), 66 to the initial level (μ=1), and only one to the developed level (μ=3). For eight records for which no abstract was available to support a reliable assessment, no μ value was assigned. The resulting distribution indicates that most applications remain at the stage of experimental research, prototyping, or limited validation, whereas mature industrial implementations are rare in the analyzed corpus.

The technology–function map also reveals clear differences among technology families. Inspection was most frequently performed using deep learning, AI/ML, and machine vision systems. Monitoring and prediction were dominated by AI/ML and deep learning, with smaller contributions from IoT and digital twins. Simulation and optimization relied primarily on AI/ML and digital twins. The audit trail function was strongly associated with blockchain, whereas data integration and documentation and reporting remained weakly represented. The most frequently observed technology–function relationships are summarized in Table 5.

The summary presents the coding results for 629 studies for which the relationship between the technology and the function performed could be determined. The reported numbers refer to the technologies most frequently occurring within a given function and do not imply that a specific technology was limited exclusively to a single type of application.

The results of Track A define the functional layer of the model. They show which technologies are associated in the literature with particular functions and how extensively these functions are represented. However, they do not yet determine the suitability of a specific technology for a particular waste-derived material. Such an assignment requires consideration of the challenges identified in Track B.

### 3.2. Results of Track B: Quality and Traceability Challenges of Waste-Derived Materials

In Track B, the search yielded a total of 701 records from the materials-oriented results of both databases. After removal of 152 duplicates, 549 records proceeded to screening. The initial selection was subsequently supplemented by verification of publication type because some review papers had been classified by the databases as regular articles or conference papers. After adopting the principle that the main Track B corpus should include primary studies only, 33 clearly secondary publications were excluded. In total, 189 records were excluded at the title and abstract screening stage, and 360 proceeded to further assessment. A further 38 publications were excluded for documented reasons, including the absence of genuine waste-derived material origin, scope outside the five material categories, a reversed direction of material use, or the absence of a quality or traceability problem. Ultimately, the main Track B corpus comprised 322 primary studies.

The synthesis of Track B was mapping oriented. For each study, the material category and the primary quality or traceability challenge were identified. The purpose of this layer was not yet to indicate technologies but to determine which problems are present in the literature concerning the individual materials. RQ1 was therefore based exclusively on the relationship:

material category×challenge

rather than on direct material–technology combinations.

Based on the adopted coding scheme, the identified problems were organized into seven operational challenge categories presented in Table 6. The categories describe the primary research problem emphasized in the publication rather than merely an individual material property. Each study was assigned one primary challenge used in the core RQ1 map, whereas secondary challenges were recorded separately. Category C4 requires particular clarification: it was applied only when control, modeling, optimization, or prediction of properties constituted the core focus of the study. Characterization of material properties alone did not provide a basis for assignment to C4.

This coding approach made it possible to compare the five material categories while maintaining a consistent rule for assigning the primary problem. Categories C1–C3 and C5–C7 refer directly to the dominant material, process, or information-related problem, whereas C4 distinguishes studies in which the predictability or controllability of properties constituted the main research focus. This distinction was particularly important for separating conventional material characterization from studies based on modeling and optimization.

**Figure 2:**
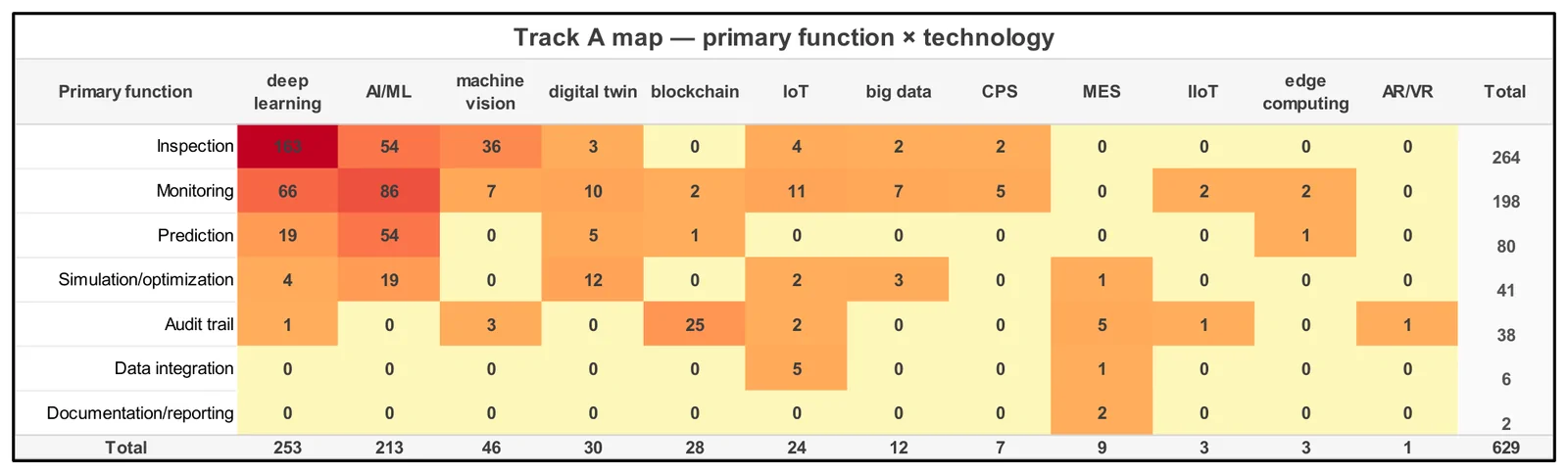
Track A map: primary function × technology (each study counted once according to its primary function). A darker shade indicates a larger number of studies.

**Figure 3:**
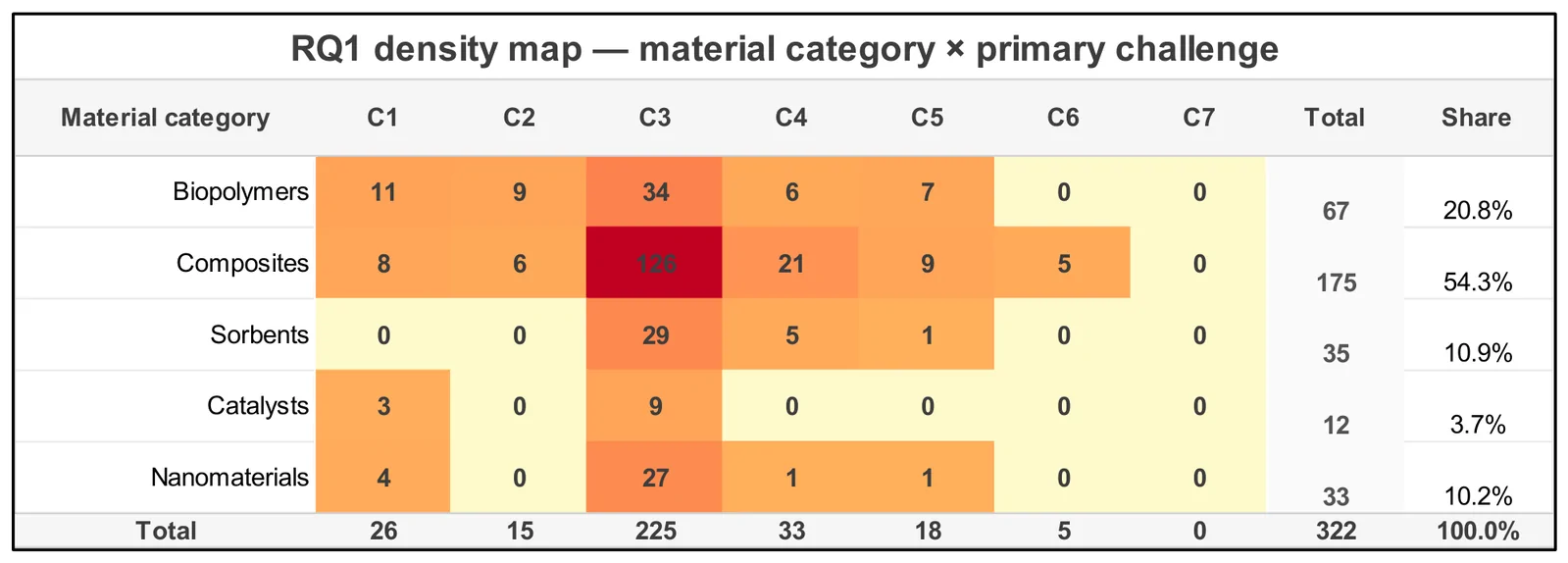
RQ1 density heatmap (material category × primary challenge). A darker shade indicates a larger number of studies.

Figure 2 presents the distribution of the primary functions of Industry 4.0 technologies in relation to the solutions identified in Track A. It shows which technologies were most frequently associated with the individual functions. Based on this taxonomy, a density map addressing RQ1 was constructed. Each of the 322 studies was counted exactly once according to its assigned material category and primary challenge. Figure 3 presents both absolute values and the shares of the individual material categories; row totals show the number of studies in each material category, whereas column totals indicate how frequently a given challenge occurred as the primary research problem across the entire corpus.

The largest single cell corresponds to the composites × C3 combination, comprising 126 studies. Thus, even at the level of absolute values, a strong concentration of the literature around microstructure, morphology, and porosity is evident, with the remaining challenge categories being represented much less extensively.

The distribution of studies in Track B was highly uneven. Of the 322 primary studies, 225 (69.9%) focused primarily on material microstructure, morphology, and porosity (C3). The second most numerous category comprised studies addressing the control and prediction of properties (C4; 33 publications, 10.2%), followed by feedstock variability and heterogeneity (C1; 26, 8.1%). Reproducibility, standardization, and scale-up (C5; 18, 5.6%), composition, purity, and contamination (C2; 15, 4.7%), and stability, durability, and degradation (C6; 5, 1.6%) were identified much less frequently as the primary challenge. Traceability, provenance, and authenticity (C7) did not occur as the primary challenge in any publication. After secondary challenges were taken into account, only two cases related to C7 were identified. The absence of this category from the primary-challenge map therefore does not indicate a complete absence of the topic, but rather its marginal position within the analyzed corpus.

For the purpose of model construction, a more detailed extraction was additionally conducted on a purposively selected sample of material situations. After secondary studies had been excluded, the sample comprised 48 situations: 22 concerning composites, 11 biopolymers, 8 sorbents, 4 nanomaterials, and 3 catalysts. Four studies concerning related methods were retained in a separate extraction section and were not counted as material situations. Quality-related problems predominated in the sample; no situation was identified in which traceability constituted the active primary requirement. This result should be taken into account when interpreting recommendations related to documentation and audit trails, because their evidence base on the Track B side remains limited.

### 3.3. Integration of Results and Model Recommendations

The two tracks were integrated through the technological function as a common level of description. Challenges identified in Track B were assigned to I4.0 functions, and the functions were then linked to technologies for which relevant applications had been identified in Track A. The model therefore does not arise from a simple comparison of publication frequencies across two independent corpora. Its starting point is a specific material situation, while the challenge–function–technology relationship provides the basis for generating candidates. For the 48 situations, the operational model matrix generated a total of 95 function–technology pairs for further evaluation. Each situation was assigned one primary function, whereas the number of candidate technologies resulted from the function–technology relationships identified in Track A.

The uneven number of situations across material categories affected the absolute number of function–technology pairs, but not the candidate-generation rule. The average number of pairs per situation was 1.95 for composites and 2.00 for each of the remaining material categories. The set of 95 pairs therefore describes the operational sample used to construct the model and is not interpreted as a representative distribution of technologies across material categories. Detailed extraction was conducted for 48 situations. The selection was purposive; therefore, the results of this sample are not used to make claims about the frequency of problems across the entire Track B corpus. The functions assigned as primary were distributed as follows: prediction, 15 situations; simulation and optimization, 15; monitoring, 10; inspection, 5; data integration, 2; and documentation and reporting, 1. This distribution describes the composition of the sample used for model construction rather than the structure of the entire materials literature. The updated sample and its distributions reflect the removal of two situations that, upon re-examination, were found to be secondary studies.

At the level of the highest-ranked result for each situation, 10 of the 48 cases received status A, 16 status B, and 22 status C. In the event of a complete tie, the status referred to the group of equivalent candidates jointly occupying the highest position. Status A denotes the presence of a directly corresponding application in the auxiliary AB set, status B denotes functional support resulting from the integration of evidence from Tracks A and B, whereas status C denotes a recommendation requiring a greater degree of analogy or extrapolation. The auxiliary AB set is not treated as a third review track. It does not contribute to answering RQ1, does not construct the technology–function map, and does not provide a basis for claiming model validation. Its role is limited to determining whether directly corresponding applications exist in the literature for selected recommendations. Accordingly, status A describes the nature of support for a recommendation rather than higher methodological quality of a study or confirmed technology effectiveness. These results provide a direct answer to RQ4. A direct counterpart in the literature was identified for 10 of the 48 top-ranked recommendations, while the remaining 38 recommendations were based on functional synthesis or required analogy or extrapolation.

In the final matrix, 40 situations resulted in a single recommendation, whereas in 8 situations two or more candidates shared the top position. Changing the weights of the individual components by ±50% did not affect the result in any of the 48 situations. Omitting the material or quality dimension changed the result in one situation each, whereas omitting the process dimension produced no changes. Using a 0–1–1 scale instead of 0–1–2 increased the number of ties in 13 situations. In the test involving local changes of ±1 point, the exact set of candidates ranked first was preserved in an average of 64.3% of the variants, while at least one candidate from the baseline result remained in first place in 81.4% of the variants. In all 48 modeled situations, quality was the only active requirement; therefore, $\sigma_{max}=6\;$ throughout the matrix. Normalization consequently did not change the ranking in any situation. The results of the sensitivity analysis are presented in Table 7. They show the extent to which changes in the adopted scoring rules affected the stability of the recommendations for the 48 analyzed situations.

Candidates were ranked primarily according to their fit to the analyzed situation. Where the σ score was the same, the literature support status e was considered, followed by the degree of grounding of the function–technology relationship, μ. A detailed formal representation of this rule is provided in Appendix A.

To illustrate the operation of the ranking procedure, Table 8 presents selected situations covering all five material categories and different types of technological functions. The summary is illustrative and does not represent the complete recommendation matrix.

In most of the cases presented, the outcome is already determined by the level of fit to the situation. A direct literature counterpart serves as additional support rather than as an automatic criterion of priority. This is particularly evident in situation s16: AI/ML and deep learning have identical values of σ, e, and μ, and therefore there is no basis for arbitrarily designating one technology as the primary solution. Both should be treated as equivalent within the boundaries of the adopted model.

The integration results also reveal a difference between functions that are widely represented in the quality-related literature and those that are less strongly represented in the materials corpus. Prediction, simulation, optimization, and monitoring can be linked to numerous quality-related situations, whereas documentation and audit trail functions have much weaker grounding on the Track B side. This does not imply that technologies such as MES, LIMS, RPA, or blockchain are inherently unsuitable. It only indicates that, for the analyzed materials, recommending them more often requires functional synthesis rather than the presence of a direct literature counterpart.

## 4. Discussion

This section interprets the integrated results, illustrates the operation of Model Ω, and discusses its limitations and practical implications.

### 4.1. Interpretation and Synthesis of the Results

The distribution observed in Track A indicates that the literature on I4.0 applications in manufacturing quality focuses primarily on process observation, nonconformity detection, and the assessment of product characteristics. Functions related to information flow, documentation, and traceability appear much less frequently. A similar imbalance was observed in Track B. The highest concentration concerns problems related to microstructure, morphology, and porosity, whereas issues of documentation, provenance, and traceability remain weakly represented. This pattern directly affects the structure of the recommendations but should not be interpreted as a simple translation of publication counts into technology suitability. The number of publications defines the extent of the existing literature base; an appropriate recommendation emerges only after the function required in a specific situation has been taken into account.

Composites showed the broadest challenge profile, including structural properties, feedstock variability, reproducibility, and durability. Their recommendations therefore focused mainly on prediction and simulation or optimization, supported by computational studies of process–structure–property relationships [23,24,25]. In biopolymers, feedstock variability, composition, and purity increased the relevance of prediction, optimization, and supporting monitoring, with direct examples including machine-learning and experimental-design approaches [26,27,28].

For sorbents and nanomaterials, recommendations were driven mainly by structural characteristics such as porosity, specific surface area, morphology, particle size, and crystallinity [29,30,31,32]. Catalysts had the smallest direct evidence base; therefore, despite documented examples of waste-derived catalyst production [33,34,35], the related technology recommendations were based primarily on indirect functional fit.

Traceability was only marginally represented in Track B, indicating that the materials literature focuses mainly on achieving and characterizing final properties rather than linking them to waste origin, batch history, and process conditions. The model may nevertheless assign data integration, documentation, and audit trail functions when traceability is an explicit requirement. Because direct material-specific evidence remains limited, the resulting recommendations, including MES, LIMS, and blockchain, should generally be treated as directions requiring further validation.

A second clear finding is the concentration of studies on microstructure, morphology, and porosity. After final corpus cleaning, this proportion was 69.9%. The predominance of this group is understandable from the perspective of material function: specific surface area and porosity determine sorbent behavior, crystallinity affects the properties of biopolymers, and morphology and particle size are of fundamental importance for nanomaterials. From the model perspective, this means that many recommendations focus on inspection, prediction, simulation and optimization. Differentiation occurs primarily at the level of the available literature support status of the recommendation and the specific process stage.

Compared with existing approaches, model Ω differs primarily in its starting point. In the analyzed studies, multi-criteria methods such as AHP, WASPAS, and MEREC were applied in individual cases to compare material alternatives or process parameters [18,19,20]. The model proposed in the present study, however, does not begin with a predefined set of alternatives. Its starting point is a situation described by the material, waste, process stage, and requirement; only on this basis is the required function determined, followed by the candidate technologies. The difference also concerns the way recommendations are ranked. In its basic variant, the model does not require the collection of differentiated criterion weights: the fit-score components are treated equally, and candidates are ranked first according to σ, then, in the event of a tie, according to the literature support status e, and subsequently according to μ. The model therefore does not replace classical MCDM methods and should not be equated with them. Its purpose is to narrow the choice space at an earlier stage and indicate technology classes justified for a specific material problem. Only at the stage of a specific implementation may multi-criteria methods be applied to compare suppliers, system variants, costs, or technical parameters.

The methodological contribution of Model Ω lies in structuring the transition from a material-specific problem to the required function and candidate technologies, while distinguishing direct, functional, and exploratory literature support. Status C, assigned to 22 of the 48 highest-ranked recommendations, indicates that at least one mapping link requires analogy, extrapolation, or relies on limited evidence. Such recommendations should be treated as transparent research hypotheses requiring validation rather than as solutions of confirmed effectiveness. The model narrows the technology-selection space but does not determine a specific product, supplier, or algorithm; these decisions require a separate assessment of feasibility, data, infrastructure, costs, and process conditions.

### 4.2. Model Application and Practical Implications

The operation of the model was illustrated using the example of a biosorbent produced from dead Pseudomonas biomass isolated from brassware workshop effluents, for which the quality requirement is to achieve high cadmium ion removal efficiency. This case was selected because it represents a relationship typical of sorbents between process parameters and sorption performance, while the analyzed literature also contains a directly corresponding study in which artificial neural networks were used to model the relationship between temperature, pH, biosorbent dosage, contact time, and process outcome [26]. This makes it possible to trace the entire recommendation mechanism and relate the model output to a direct literature counterpart. The full formal representation of the example is provided in Appendix A.4.

Unlike the operational matrix of 48 situations, in which each situation was assigned one primary function, the example deliberately presents an extended variant of the model that also includes a supporting function. This illustrates the possibility of constructing a complementary set of solutions for a single material problem.

The input situation was described in accordance with Equation (1), assuming:

(3) $$\begin{array}{l} m=\mathrm{sorbent}\\ w=dead\;Pseudomonas\;biomass\;from\;industrial\;effluents\\ p=sorption\;application\\ q=target\;Cd\;ion\;removal\;efficiency \end{array}$$

Because sorption efficiency is an outcome dependent on process conditions, prediction was assigned as the primary function. Its purpose is to predict the process response based on input parameters such as temperature, pH, biosorbent dosage, and contact time. Monitoring was treated as a supporting function related to recording the parameters that influence the course of the sorption process. The mapping of the situation to functions therefore takes the form:

(4) Φ(s)={prediction,monitoring}

Candidate technologies were then identified for each function based on the technology–function map obtained in Track A. Under the adopted coding scheme, AI/ML and deep learning were the most frequently occurring solutions for prediction; an analogous procedure was applied to the monitoring function. Each function–technology pair was assigned a recommendation literature support status e, a fit score σ, and an indicator μ.

For the combination of prediction and AI/ML, a directly corresponding literature application was identified. In the analyzed study, artificial neural networks were used to model the cadmium sorption process while accounting for temperature, pH, biosorbent dosage, and contact time. The recommendation was assigned status A. The remaining solutions had an indirect or exploratory basis, depending on whether both mapping links, the challenge–function relationship and the function–technology relationship, were independently supported in the literature. The ranking of candidate technologies for the analyzed situation is presented in Table 9.

The value of σ results from four fit components: fit to the material properties, process stage, quality requirement, and traceability requirement. For the prediction function, full fit to the active quality requirement was assumed, whereas the monitoring function received a lower score for this component because it supports the control of process parameters but does not directly predict sorption efficiency. The indicator μ is derived from the Track A coding and, in the analyzed example, corresponds to the emerging level.

The highest-ranked solution was AI/ML applied to the prediction of sorption-process efficiency. The outcome resulted from the combination of a direct literature counterpart and a high fit to the situation. Deep learning was retained as an alternative solution because it performs the same prediction function but lacks equally direct support for the analyzed case. Monitoring serves a supporting role: it can provide data on process conditions that subsequently feed the predictive model. A deep-learning-based solution for the monitoring function remains an exploratory proposal.

The example also illustrates the roles of the individual model layers. Track B provides a description of the material problem and the basis for assigning the required function, whereas Track A identifies technologies capable of performing that function and the degree to which their function–technology relationships are grounded in the literature. The recommendation was assigned status A because a directly corresponding application of artificial neural networks for the analyzed material situation was identified in the auxiliary AB set.

### 4.3. Limitations and Further Validation

The first limitation of the study concerns the way the corpora were constructed. In Track B, a materials-oriented subset of the results was used, which increased the feasibility of screening and consistency with the scope of the study, but may have limited the representation of publications from the fields of chemistry, chemical engineering, environmental sciences, and energy. The impact of this restriction was assessed against a broader reference corpus; however, the comparative analysis itself does not eliminate the risk of omitting relevant studies. The model output should therefore be interpreted within the limits of the adopted search strategy.

The second limitation results from the level of data extraction. The mapping layer was constructed mainly on the basis of titles, abstracts, and keywords, whereas full texts were analyzed in cases requiring in-depth assessment and in the sample used to construct the model situations. This approach made it possible to analyze a larger corpus, but may have led to the omission of information described exclusively in the methodological or results sections of the articles. An additional limitation is that the coding was conducted by a single researcher, which prevented a quantitative assessment of inter-rater agreement.

The third limitation concerns the purposive selection of situations used for in-depth extraction. Priority was given to studies that made it possible to reconstruct the relationships between the material, the problem, and the technology, including applications of computational methods. This selection increases the usefulness of the sample for model construction, but may overrepresent cases in which a direct relationship with a digital solution is already visible. Separating the full corpus map from the extraction sample reduces this risk, but does not eliminate it. Consequently, the 95 function–technology pairs should be interpreted as the output of the operational sample rather than as a representative distribution of technology recommendations across all five material categories. No formal assessment of methodological quality or risk of bias was performed. Consequently, the frequencies reported in Track A reflect the representation of technology–function relationships in the publication corpus and should not be interpreted as the strength of evidence, implementation effectiveness, or likelihood of successful deployment.

Another limitation concerns the fit score σ. Although an explicit rubric for material, process, and functional assessments was applied, the fit values include an element of authorial judgment. The literature support status e was separated from the fit score; however, further calibration of the model should include independent expert assessment or comparison with implementation results. The operational analysis of 48 situations used one primary function assigned to the dominant challenge. This facilitated comparison between situations and limited the size of the matrix, but may have omitted cases requiring the simultaneous performance of several functions. The extended multi-function variant was illustrated in the example, but was not systematically applied to all situations.

Another limitation is associated with the indicator μ. Its values in the analyzed Track A are strongly concentrated around a single level; therefore, the ability of this indicator to differentiate recommendations is limited. Moreover, μ should be interpreted as the degree of grounding of the function in the analyzed literature, rather than as general technological maturity or implementation readiness. In the operational matrix, 94 of the 95 candidates had μ=2, and the indicator did not affect the ordering in any situation; thus, in the present application, the ranking rule σ→e→μ operated in practice as σ→e.

The sensitivity analysis showed that the model results were robust to moderate changes in the weights of the individual components of σ. At the same time, flattening the scale to the 0–1–1 variant increased the number of ties, indicating that distinguishing between indirect and direct fit improves the model’s ability to differentiate between candidates. Greater sensitivity was observed when individual fit scores were changed locally, particularly in situations where technologies had similar levels of fit. The equal weights were therefore retained, while the fit scores themselves require further calibration by experts or comparison with the outcomes of real-world implementations.

A potential source of bias is also the operational mapping Ψ, in which candidate technologies are selected based on their frequency of co-occurrence with a function in Track A. This approach increases reproducibility but favors technologies that are widely represented in the literature, particularly AI/ML and deep learning. Consequently, less frequent solutions that may nevertheless be well fitted to a specific situation may not enter the narrow candidate set. Sensitivity analysis should therefore include varying the number of technologies assigned to a function and comparing results based on absolute counts and relative shares. The technology taxonomy itself also requires additional consistency. The AI/ML and deep learning categories remain hierarchically related because deep learning is part of machine learning. The two categories were nevertheless retained as separate operational coding labels reflecting the terminology used in the source publications and should not be interpreted as taxonomically independent technology classes. If both categories are retained as separate coding units, AI/ML should be consistently treated as a category comprising solutions other than deep learning, or a hierarchical classification level should be introduced. The absence of such a distinction may lead to partial double representation of similar solutions.

Another limitation is the static and unidirectional nature of the model. The current procedure leads from problem definition to technology recommendation but does not incorporate feedback from implementation. This is consistent with the model’s current purpose as a tool supporting the initial selection of technologies. In future research, the model should be extended to include a feedback loop that would allow recommendations to be updated based on the outcomes of real-world applications.

The most important limitation remains the lack of independent external validation. The literature support status of the recommendations, the fit score, and the indicator μ are elements of the model construction and cannot themselves confirm its validity. Internal consistency checking makes it possible to assess whether a recommendation follows from the declared rules, but it does not answer the question of whether the technology will prove effective in a new material situation. Further validation should therefore include applying the model to cases not used in its construction. The literature support status does not account for the number or methodological quality of supporting studies. Consequently, status A should not be interpreted as a higher level of evidence than status B, but only as a more direct correspondence between the publication and the analyzed decision situation.

In none of the 48 model situations did traceability constitute an active primary requirement. This means that the branch of the model concerning documentation, data integration, and the audit trail was not empirically applied to the same extent as the quality-related functions. This limitation corresponds to the structure of Track B, in which C7 did not occur as a primary challenge and, after secondary challenges were taken into account, only two cases were recorded. Therefore, solutions related to data integration, documentation, and the audit trail should be treated as implementation hypotheses requiring verification. Their value may be assessed, among other aspects, through their ability to link a material batch with the waste source, documentation completeness, the possibility of reconstructing process history, and the time required to identify the causes of nonconformities. Model Ω is therefore not a tool that guarantees the optimal selection of technology. Its role is to structure the decision-making process and indicate solutions that are consistent with the nature of the problem and have a specified status of literature support. Implementation of the recommendations requires further assessment of feasibility, data availability, integration with existing infrastructure, and validation under the conditions of a specific process.

## 5. Conclusions

The aim of the study was to develop a model supporting the selection of I4.0 technologies for quality assurance and traceability in the production of waste-derived functional materials. The proposed model Ω links a decision situation described by the material category, waste type, process stage, and quality or traceability requirement with the required I4.0 function and subsequently with technologies capable of performing that function. Each recommendation is additionally described by the literature support status e, the fit score σ, and the indicator μ, which refers to the degree of grounding of the function–technology relationship in the analyzed literature.

The model was developed based on the results of a two-track scoping review, supplemented by an auxiliary set of direct cases. Track A provided the technology–function map, while Track B enabled the identification and organization of challenges across five categories of waste-derived functional materials: biopolymers, composites, sorbents, catalysts, and nanomaterials. The integration of both layers made it possible to generate and rank technology recommendations.

The analysis of Track B revealed a strong thematic concentration. Of the 322 primary studies, 225 (69.9%) concerned primarily material microstructure, morphology, and porosity (C3). The second most numerous category comprised control and prediction of properties (C4; 33 studies, 10.2%), followed by feedstock variability and heterogeneity (C1; 26 studies, 8.1%). Traceability, provenance, and authenticity (C7) did not occur as a primary challenge in any publication; after secondary challenges were taken into account, only two cases were recorded. The most extensively represented material group was composites, comprising 175 of the 322 studies (54.3%), followed by biopolymers with 67 (20.8%), sorbents with 35 (10.9%), nanomaterials with 33 (10.2%), and catalysts with 12 (3.7%).

On the technology side, the analysis of 629 studies allowing unambiguous mapping of technology–function relationships showed the predominance of inspection (264 studies; 42.0%) and monitoring (198; 31.5%). Prediction comprised 80 studies (12.7%), simulation and optimization 41 (6.5%), and audit trail 38 (6.0%). Data integration (6; 1.0%) and documentation and reporting (2; 0.3%) remained marginal. At the same time, 554 of the 629 mappable studies (88.1%) were assigned to the emerging grounding level μ=2, whereas only one study (0.2%) reached level μ=3. These results indicate that the analyzed literature is dominated by experimental and prototype applications, with limited representation of developed operational implementations.

In the in-depth sample of 48 model situations, prediction and simulation and optimization predominated, with 15 situations each (31.3%), followed by monitoring with 10 (20.8%) and inspection with 5 (10.4%). Data integration occurred in two situations (4.2%), and documentation and reporting in one (2.1%). The operational matrix comprised 48 situations and 95 function–technology pairs. At the level of the highest-ranked result for each situation, 10 cases (20.8%) had status A, 16 (33.3%) status B, and 22 (45.8%) status C. The sensitivity analysis confirmed that the recommendations were stable with respect to changes in the weights, while cases involving ties and closely matched scores were more sensitive to one-point changes in the fit scores. With respect to RQ4, this distribution indicates that only some of the recommendations had direct support in the literature, whereas most were derived from functional synthesis or were exploratory in nature. In situations involving a complete tie, the highest position was jointly occupied by equivalent candidates.

The integration of the two tracks showed that the direct literature basis for the recommendations was uneven. In cases where publications directly linked the material situation with the technology, the recommendation was assigned status A. Where the literature independently supported the relationship between the challenge and the function and between the function and the technology, status B was applied. Status C was reserved for proposals based on analogy or limited functional support. This distinction makes it possible to answer not only which technology may be useful, but also on what basis it was indicated and where the direct evidence ends.

Traceability was weakly represented in the evidence base. Category C7 did not occur as a primary challenge in Track B, and none of the 48 modeled situations included traceability as an active primary requirement. Consequently, recommendations concerning data integration, documentation, audit trails, MES, LIMS, and blockchain should be interpreted as exploratory directions requiring further validation rather than as solutions of confirmed effectiveness.

The main methodological contribution of the study lies in systematizing the transition:

material challenge→I4.0 function→technology

and in explicitly separating directly documented recommendations from indirect and exploratory ones. The separation of the full mapping layer from the in-depth extraction of model situations reduces the risk of equating the frequency of a problem in the literature with the strength of a technology recommendation. However, it does not completely eliminate the influence of the adopted guidelines, sample selection, or mapping rules.

The model has several limitations. The materials-oriented filter applied in Track B may have limited the representation of part of the literature published outside the main field of Materials Science. A substantial part of the mapping was performed on the basis of titles, abstracts, and keywords, which may have led to the omission of information available only in full texts. The sample used for in-depth extraction was purposive rather than random and may therefore have overrepresented cases with a clear computational component. The indicators μ and σ also showed limited discriminatory ability, while the technology-selection rule in the Ψ mapping favored solutions widely represented in the literature. The model has not yet undergone independent external validation and should therefore be treated as a decision–support tool rather than as a validated indicator of optimal technology selection.

Industry 4.0 provides the technological foundation addressed by Model Ω, whereas Industry 5.0 complements it with sustainability, resilience, and human-centricity. Industry 6.0 remains an emerging and inconsistently defined concept, without a sufficiently established and empirically validated framework to justify reclassifying the model. Model Ω therefore retains its Industry 4.0 grounding, while the broader criteria associated with later industrial paradigms are treated as directions for future extension.

Further research should focus primarily on independent validation of the model. This includes applying Ω to new material situations not used in its construction, comparing the recommendations with solutions observed in practice, and assessing the stability of the results when the technology-selection rules and the method of calculating σ are changed. An important direction is also to extend the analysis to areas that are weakly represented in the current corpus and to examine whether the use of broader search strategies changes the structure of the identified challenges. One possible area for future extension is waste-derived construction materials; however, their applicability within the model should be assessed separately using construction-specific quality, durability, and environmental requirements. The next stage should be validation in an industrial environment. It would be particularly valuable to examine whether recommendations concerning data integration, documentation, and the audit trail translate into measurable improvements in material traceability, documentation completeness, the ability to reconstruct batch history, and the time required to determine the causes of nonconformities. Only such applications will make it possible to assess the extent to which the model can support actual technology decisions in the production of waste-derived functional materials.

## Figures and Tables

**Table 1:** Research directions related to waste-derived functional materials, I4.0 technologies, and decision support.

Study	Main Scope	Contribution Relevant to the Present Study	Scope Not Addressed
[3]	waste-derived functional materials	material classes and their functional properties	no model for selecting I4.0 technologies for quality-related problems
[7,8]	material passports and digital product passports	traceability, provenance, and information flows in the circular economy	no linkage between technology selection and the properties of a specific waste-derived material
[12]	automation, supervisory systems, and I4.0	architectures and functions of digital technologies in industrial environments	no consideration of the specific characteristics of waste-derived functional materials
[13]	AI/ML in quality assurance	quality prediction and applications of machine learning methods	no multi-technology selection model dependent on material type and waste source
[15]	digital twins	process representation, simulation, and optimization	analysis of a single technology class, without a comparative model for waste-derived materials
[16,17]	LIMS and RPA	data integration, documentation, and automation of information flows	no linkage to quality challenges specific to particular materials
[18,19,20]	MCDM and selection of materials/material alternatives	formal decision support for waste-derived materials	selection concerns materials or alternatives rather than I4.0 technologies for quality and traceability

**Table 2:** Functional layers of Industry 4.0 technologies.

Layer	Model Functions	Example Technologies	Methodological Note
Process control and monitoring	monitoring	IoT, SCADA, MES	primarily concerns manufacturing processes and waste-feedstock processing conditions
Quality analysis and prediction	prediction	AI/ML, predictive models, digital twin	uses laboratory and process data
Material inspection	inspection	machine vision systems, image analysis, AI	directly relates to the assessment of material characteristics
Process simulation and optimization	simulation/optimization	digital twin, cyber–physical systems, AI	requires a process model and may use monitoring data
Data integration	data integration	MES, big data, edge computing, blockchain	integrates process, laboratory, and quality data
Traceability and documentation	documentation/reporting; audit trail	RPA, LIMS, ERP/MES, potentially blockchain	RPA primarily serves a supporting function and is not a technology for measuring material properties

**Table 3:** Data Sources and Hierarchical Structure of the Datasets Used to Develop the Model.

Level of Analysis	Sources and Scope	Number of Records	Unit of Analysis	Role in the Model
Track A: technology corpus	Scopus and Web of Science; high-precision searches in publication titles; journal articles, conference papers, and review papers concerning the functions of I4.0 technologies in quality and traceability	1220 records; 752 after deduplication; 718 studies included; 629 studies with an unambiguous technology–function relationship	Publication	Development of the technology–function map and determination of the degree to which each relationship is grounded in the literature
Track B: material corpus	Scopus and Web of Science; searches in titles, abstracts, and keywords; material-specific subsets; primary studies only	701 records; 549 after deduplication; 322 studies included	Publication	Identification and mapping of challenges C1–C7 across five categories of waste-derived functional materials
Track B: in-depth extraction	Purposively selected sample of studies from the Track B corpus	48 material situations	Situation *s* = <*m*,*w*,*p*,*q*>	Identification of the material category, waste source, process stage, and requirement, and assignment of the required I4.0 function
Model integration level	Integration of situations from Track B, function–technology relationships from Track A, and direct cases from the AB set	48 situations; 95 function–technology pairs	Candidate (*f*,*t*) for situation *s*	Evaluation of fit, the literature support status, and relationship grounding, followed by the generation of the ranked recommendation *Ω*(*s*)

**Table 4:** Literature support status of recommendations.

Status	Nature of Support	Meaning
A	direct literature counterpart	a publication corresponding to the analyzed situation and the recommended technology was identified in the auxiliary AB set
B	functional support	no direct counterpart was identified in the AB set, but the challenge is documented in Track B and the function–technology relationship in Track A
C	exploratory recommendation	at least one mapping link requires analogy or extrapolation, or has limited support

**Table 5:** Most frequently observed technology–function relationships in Track A.

Function	Dominant Technologies	Number of Studies
Monitoring	AI/ML (86), deep learning (66), IoT (11), digital twins (10)	198
Prediction	AI/ML (54), deep learning (19), digital twins (5)	80
Inspection	deep learning (163), AI/ML (54), machine vision (36)	264
Simulation/optimization	AI/ML (19), digital twins (12), deep learning (4)	41
Data integration	IoT (5), MES (1)	6
Documentation/reporting	MES (2)	2
Audit trail	blockchain (25), MES (5), machine vision (3)	38

**Table 6:** Taxonomy of quality and traceability challenges in Track B.

Code	Challenge	Scope
C1	Feedstock variability and heterogeneity	differences related to waste source, batch, season, location, and heterogeneity
C2	Composition, purity, and contamination	chemical composition, purity, presence of contaminants and trace elements
C3	Microstructure, morphology, and porosity	morphology, particle size, structure, porosity, and specific surface area
C4	Control and prediction of properties	modeling, optimization, and process–structure–property relationships
C5	Reproducibility, standardization, and scale-up	batch-to-batch reproducibility, standardization of procedures, and scale-up
C6	Stability, durability, and degradation	service durability, degradation, biodegradation, leaching, and fouling
C7	Traceability, provenance, and authenticity	tracking material origin and history, and linkages along the chain

**Table 7:** Results of the Sensitivity Analysis of the σ Score.

Analysis Variant	Effect on the Model Output
Baseline 0–1–2 variant	40 single recommendations; 8 ties
Change in an individual weight by ±50%	No changes across the 48 situations
Omission of the material dimension	Change in 1 of the 48 situations
Omission of the process dimension	No changes
Omission of the quality dimension	Change in 1 of the 48 situations
0–1–1 scale	Changes in 13 situations due to the reappearance of ties
Local changes in fit by ±1	The exact result was preserved in an average of 64.3% of the variants; at least one original top-ranked candidate remained in 81.4% of the variants
$\mathrm{Normalized}\;\mathrm{score}\;\sigma_{N}=\sigma /\sigma_{max}$	No change in the ranking of the 48 situations

**Table 8:** Representative excerpt from the decision matrix.

Situation	Category	Function	Technology	e	σ	μ	Role
s13	Composites	simulation/optimization	AI/ML	A	6	2	primary
		simulation/optimization	digital twin	C	3	2	exploratory
s121	Biopolymers	prediction	AI/ML	A	6	2	primary
		prediction	deep learning	C	3	2	exploratory
s232	Sorbents	simulation/optimization	AI/ML	A	6	2	primary
		simulation/optimization	digital twin	C	3	2	exploratory
s16	Catalysts	monitoring	AI/ML	C	3	2	equivalent
		monitoring	deep learning	C	3	2	equivalent
s321	Nanomaterials	simulation/optimization	AI/ML	B	5	2	primary
		simulation/optimization	digital twin	C	3	2	exploratory
s77	Composites	data integration	IoT	A	5	2	primary
		data integration	MES	C	3	2	exploratory

**Table 9:** Technology ranking for the example material situation.

Rank	Function	Technology	e	σ	μ	Role
1	prediction	AI/ML	A	6	2	primary
2	prediction	deep learning	B	6	2	alternative
3	monitoring	AI/ML	B	5	2	supporting
4	monitoring	deep learning	C	5	2	exploratory

## Data Availability

The original contributions presented in the study are included in the article, further inquiries can be directed to the corresponding author.

## Abbreviation

The following abbreviation is used in this manuscript:

I4.0	Industry 4.0

## Appendix A. Formalization of the Decision–Support Model

The Appendix presents the formal representation of the model described in the methodological section. Its purpose is to structure the transition from the material situation to the required Industry 4.0 function and subsequently to the technology recommendation. The formalization serves to ensure transparency and reproducibility of the reasoning; it does not constitute a separate optimization algorithm.

The general logic of the model can be represented as:

material situation→I4.0 function→technology→ordered recommendation

### Appendix A.1. Decision Situation

The input unit of the model is a situation requiring a technology decision, described as:

(A1) s=⟨m,w,p,q⟩

where

m—denotes the functional material category;

w—the waste type or source;

p—the process stage;

q—the quality or traceability requirement, or a combination thereof.

The set of material categories comprises the five groups adopted in the study:

(A2) M={biopolymers,composites,sorbents,catalysts,nanomaterials}

### Appendix A.2. Mapping the Situation to a Function and Technology

The set of I4.0 technology functions is denoted as:

(A3) F={monitoring,prediction,inspection,simulation/optimization,data integration,documentation/reporting,audit trail}

The first mapping of the model assigns one or more functions to a situation:

(A4) $$\Phi :S\to 2^{F}$$

The assignment of functions is based on the type of challenge and the process stage. It is therefore not performed solely on the basis of an unconstrained assessment of an individual case.

For example:

- feedstock variability and heterogeneity may require monitoring and prediction;
- problems related to composition, purity, or contamination may lead to monitoring and inspection;
- morphology, porosity, and specific surface area may require inspection, prediction, or simulation and optimization;
- requirements concerning material provenance may lead to data integration, documentation, and maintenance of an audit trail.

Detailed transition rules between challenge categories and functions result from the guidelines adopted at the stage of synthesis of the two tracks.

The second mapping links a function with technologies:

(A5) $$\Psi :F\to 2^{T}$$

where T denotes the set of technologies identified in Track A. The relation Ψ is determined on the basis of the empirical technology–function map. In the operational version of the model, for each function, the technologies with the highest representation in Track A are considered; in the basic variant, the two most frequently occurring technologies for a given function were adopted. This fixed-size rule was adopted to limit the expansion of the candidate set and to ensure a comparable operational assessment across situations; it should not be interpreted as an exhaustive selection of all potentially suitable technologies.

For a situation s, the candidate set takes the form:

(A6) C(s)={(f,t):f∈Φ(s),t∈Ψ(f)}

This set does not yet constitute the final recommendation. It only defines the possible function–technology pairs that proceed to further evaluation.

### Appendix A.3. Evaluation and Ranking of Recommendations

Each candidate pair is described by three distinct characteristics:

- fit to the situation σ;
- literature support status e;
- degree of grounding of the function–technology relationship μ.

#### Appendix A.3.1. Fit to the Situation

The score σ determines the compatibility of the function–technology pair with the analyzed situation. Four possible dimensions are assessed:

- material property;
- process stage;
- quality requirement;
- traceability requirement.

For each active dimension, a value is assigned:

(A7) $$fit_{d}\in \{0,1,2\}$$

where 0 denotes no fit, 1 partial fit, and 2 full fit. The total score is:

(A8) $$\sigma (s,f,t)=\sum_{d}fit_{d}(s,f,t)$$

For comparisons between decision situations, the normalized score is defined as:

(A9) $$\sigma_{N}(s,f,t)=\frac{\sigma (s,f,t)}{\sigma_{max}(s)}.$$

The value of $\sigma_{N}$ ranges from 0 to 1. Since $\sigma_{max}(s)$ is common to all candidates within a given situation, normalization does not affect their ranking.

The quality or traceability component is included only when the corresponding requirement is active in the given situation. For this reason, σ is used primarily to rank candidates within the same decision situation. Results obtained for situations with different numbers of active requirements are not treated as directly comparable.

#### Appendix A.3.2. Literature Support Status

The status:

(A10) e∈{A,B,C}

describes the nature of the literature support for the recommendation.

A—direct confirmation.

At least one publication directly corresponding to the analyzed material situation and the application of the recommended technology was identified in the auxiliary AB set. The function may be reconstructed from the content of the publication even if it was not explicitly named.

B—functional support.

No direct counterpart was identified in the AB set, but both links of the synthesis have literature support: Track B documents the occurrence of the given material challenge, whereas Track A documents the capability of the technology to perform the required function.

C—exploratory recommendation.

At least one mapping link is based on analogy, functional extrapolation, or limited literature support.

Status A/B/C does not constitute an assessment of the methodological quality of publications or validation of technology effectiveness. It serves exclusively to distinguish recommendations having a directly corresponding literature example from recommendations based on functional synthesis or extrapolation.

The auxiliary AB set does not constitute a separate review track. It is not used to answer RQ1 or to construct the technology–function map in Track A. Its role is limited to checking whether directly corresponding applications reported in the literature exist for selected recommendations.

#### Appendix A.3.3. Degree of Grounding of the Function–Technology Relationship

The indicator:

(A11) μ(f,t)∈{1,2,3}

describes the degree of grounding of the function–technology relationship in the Track A literature:

μ=1

− early, conceptual, or weakly represented application;

μ=2

− emerging, experimentally verified, or prototype application;

μ=3

− developed application, confirmed in an operational or industrial environment.

The indicator μ is not equated with the Technology Readiness Level (TRL) or with the overall market maturity of the technology. Its meaning is limited to the degree of grounding of the analyzed relationship in the Track A corpus.

#### Appendix A.3.4. Ranking Rule

Recommendations are ranked according to the sequence:

(A12) σ→e→μ

This means that priority is given to the candidate with the best fit to the analyzed situation. If two or more candidates obtain the same σ score, the recommendation with the stronger literature support status is ranked higher, following the order A, B, C. The indicator μ serves as the next auxiliary criterion.

The output of the model is an ordered list of candidates:

(A13) $$\Omega (s)=\langle c_{\left(1\right)},c_{\left(2\right)},\ldots ,c_{\left(n\right)}\rangle$$

A unique candidate occupying the highest position is treated as the primary recommendation, whereas candidates with identical values of σ, e, and μ are treated as equivalent. Complementary solutions performing an additional function may be designated as supporting. Status C indicates the exploratory nature of the literature support but does not preclude a candidate from occupying the highest position in a situation for which no better-fitted and more strongly supported candidate has been identified.

### Appendix A.4. Application Example

The operation of the model is presented for the case of a biosorbent produced from dead *Pseudomonas* biomass isolated from brassware workshop effluents. The material was used for the removal of cadmium ions, and in a directly corresponding study, the dependence of the process outcome on temperature, pH, biosorbent dosage, and contact time was modeled using artificial neural networks [25]. The example corresponds to the case discussed in Section 4.1 and serves to demonstrate the complete transition from the material situation to an ordered technology recommendation.

The input situation was defined in accordance with Equation (A1), assuming:

(A14) $$\begin{array}{cc} m & =\mathrm{sorbent}\\ w & =\mathrm{dead}\;\mathrm{Pseudomonas}\;\mathrm{biomass}\;\mathrm{from}\;\mathrm{industrial}\;\mathrm{effluents}\\ p & =\mathrm{sorption}\;\mathrm{application}\\ q & =\mathrm{target}\;\mathrm{Cd}\;\mathrm{ion}\;\mathrm{removal}\;\mathrm{efficiency} \end{array}$$

The requirement q is quality related. It concerns prediction of the sorption process response depending on the input parameters; therefore, prediction was assigned as the primary function. In the extended variant of the example, monitoring was also included as a supporting function related to the recording of parameters affecting the course of the process. Thus, the following was obtained:

(A15) Φ(s)={prediction,monitoring}

For each function, candidate technologies were identified on the basis of the technology–function relationships identified in Track A. For the analyzed example, the following were adopted:

(A16) Ψ(prediction)={AI/ML,deep learning}Ψ(monitoring)={AI/ML,deep learning}

As a result of combining the two mappings, four candidate pairs were obtained:

(A17) C(s)={(prediction,AI/ML),(prediction,deep learning),(monitoring,AI/ML),(monitoring,deep learning)}

Each pair was assigned a literature support status e, a fit score σ, and an indicator μ. For the combination of prediction and AI/ML, a directly corresponding literature application was identified in which artificial neural networks were used to model the cadmium sorption process for the corresponding material situation [25]. For this reason, the recommendation was assigned status A.

For deep learning in the prediction function, no equally direct counterpart was identified; however, Track B documents the analyzed material problem, whereas Track A documents the relationship between prediction and deep learning. Status B was therefore assigned. For the monitoring function, the combination with AI/ML received status B, whereas the combination with deep learning received status C. The set of statuses takes the form:

(A18) $$\begin{array}{cc} e(s,\mathrm{prediction},\mathrm{AI}/\mathrm{ML}) & =A,\\ e(s,\mathrm{prediction},\mathrm{deep}\;\mathrm{learning}) & =B,\\ e(s,\mathrm{monitoring},\mathrm{AI}/\mathrm{ML}) & =B,\\ e(s,\mathrm{monitoring},\mathrm{deep}\;\mathrm{learning}) & =C. \end{array}$$

The fit score was determined independently of status e. For both candidates performing the prediction function, full fit to the material category, process stage, and active quality requirement was assumed:

(A19) $$\begin{array}{cc} fit_{\mathrm{material}} & =2,\\ fit_{\mathrm{process}} & =2,\\ fit_{\mathrm{quality}} & =2,\\ fit_{\mathrm{traceability}} & =0. \end{array}$$

Thus:

(A20) σ=2+2+2+0=6.

For the monitoring function, a lower fit to the active quality requirement was assumed because the recording of process parameters may support the predictive model but does not itself predict sorption efficiency:

(A21) $$\begin{array}{cc} fit_{\mathrm{material}} & =2,\\ fit_{\mathrm{process}} & =2,\\ fit_{\mathrm{quality}} & =1,\\ fit_{\mathrm{traceability}} & =0. \end{array}$$

which gives:

(A22) σ=2+2+1+0=5.

For all four pairs, the indicator μ took the value 2, corresponding to the emerging level of the function–technology relationship in Track A. The candidate ranking therefore took the form:

(A23) $$\Omega (s)=\left[\begin{array}{c} (prediction,AI/ML,A,6,2),\\ (prediction,deep\;learning,B,6,2),\\ (monitoring,AI/ML,B,5,2),\\ \left(\mathrm{monitoring},\mathrm{deep}\;\mathrm{learning},C,5,2\right) \end{array}\right].$$

The candidates were ranked according to the rule defined in Equation (A12):

σ→e→μ.

The highest position was occupied by the pair:

(prediction,AI/ML),

because, with the maximum fit score σ=6, it had status A. The pair:

$$\left(\mathrm{prediction},\mathrm{deep}\;\mathrm{learning}\right)$$

obtained the same fit score but a lower literature support status B and was therefore classified as an alternative solution performing the same function. The pair:

$$\left(\mathrm{monitoring},\mathrm{AI}/\mathrm{ML}\right)$$

serves a supporting role because it performs a separate function complementary to prediction. The final pair is exploratory because of its status C.

The example shows that the position of a technology does not result solely from its presence in the literature. The final ranking is determined first by fit to the situation σ, then by the nature of the literature support e, and, in the event of a further tie, by the degree of grounding of the function–technology relationship μ. The direct application of artificial neural networks in a corresponding material situation makes it possible, in this case, to assign status A to the AI/ML recommendation, but does not in itself replace the fit assessment performed within the model.

The example demonstrates the complete logic of the model:

(A24) s→Φ(s)→Ψ(f)→(σ,e,μ)→Ω(s)

The presented example therefore illustrates the complete transition from the description of the material situation, through the identification of the required function and candidate technologies, to an ordered recommendation that takes into account fit, the nature of the literature support, and the degree of grounding of the function–technology relationship.
